# Medication Effects on Heart Rate Variability in Critical Illness: The Overlooked Confounder

**DOI:** 10.1097/CCE.0000000000001386

**Published:** 2026-03-04

**Authors:** Kelli Henry, Brian Murray, Rishikesan Kamaleswaran, Susan E. Smith, Emily Grace Moore, Kaitlin Blotske, Andrea Sikora

**Affiliations:** 1 Department of Biomedical Informatics, University of Colorado Anschutz, Aurora, CO.; 2 Department of Clinical Pharmacy, Skaggs School of Pharmacy and Pharmaceutical Sciences, University of Colorado Anschutz Medical Campus, Aurora, CO.; 3 Department of Surgery, Duke University, Durham, NC.; 4 Department of Clinical and Administrative Pharmacy, University of Georgia College of Pharmacy, Athens, GA.

**Keywords:** critical care, heart rate variability, medications, sepsis, shock

## Abstract

Heart rate variability (HRV) reflects autonomic nervous system function and has emerged as a potential noninvasive biomarker for early detection of physiologic deterioration in critical illness. HRV-based prediction models show promise; however, translation into routine ICU practice has been limited. A major barrier is the insufficient characterization of medication effects on HRV. Pharmacologic agents commonly used in critical care, including vasopressors, steroids, and antiarrhythmics, can directly or indirectly alter autonomic tone, yet existing studies rarely account for these influences. As a result, medication-induced HRV changes may represent meaningful therapeutic response or misleading confounding noise, complicating interpretation. Current studies do not adequately account for medication exposure when evaluating HRV in critical illness. We outline research priorities focused on quantifying medication effects, integrating medication exposure into predictive modeling, evaluating HRV as a marker of treatment response, and determining the utility of HRV as a treatment target.

Heart rate variability (HRV), defined as the variation in time intervals between consecutive heartbeats, is a surrogate marker of autonomic nervous system function. Clinically, low HRV reflects sympathetic predominance and physiologic stress and has been associated with increased illness severity and mortality in critical illness. In contrast, higher HRV generally reflects preserved vagal modulation and greater physiologic adaptability. HRV is quantified using time-domain measures (e.g., the sd of NN intervals, the root mean square of successive differences) and frequency-domain measures (e.g., high-frequency [HF] power reflecting parasympathetic activity, low-frequency [LF] power reflecting mixed sympathetic and parasympathetic influences, and the LF/HF ratio).

Alterations in HRV are associated with autonomic dysregulation and impaired homeostatic control, generating interest in HRV as an early noninvasive biomarker for sepsis and critical illness (1–3). HRV-based machine learning models have shown promise for predicting sepsis-associated acute respiratory failure and septic shock (4–6), and limited evidence suggests that earlier identification of physiologic deterioration using HRV may inform timely intervention and reduce mortality (7). However, clinical trials evaluating HRV monitoring have produced inconsistent results and real-world adoption remains limited by an incomplete mechanistic understanding of the factors that modulate HRV in the ICU environment. Among these factors, medication therapy remains the most under-characterized and clinically consequential confounder (2, 8–10).

Pharmacologic agents commonly used in critical care, such as vasopressors, steroids, and antiarrhythmics, modulate sympathetic and parasympathetic tone either directly or indirectly through treatment response and may therefore influence HRV indices. However, the magnitude, directionality, consistency, and relevance of these effects are incompletely understood. This gap in understanding confounds interpretation of HRV parameters in critically ill patients receiving complex medication regimens (11). This commentary summarizes the current evidence regarding HRV monitoring in critically ill patients, highlights the limited evaluation of medication effects in existing studies, and outlines actionable research priorities to improve the interpretability and clinical utility of HRV in the ICU.

## CURRENT EVIDENCE AND LIMITATIONS

To understand how prior work addressed medication exposure, a literature review was performed based on the Preferred Reporting Items for Systematic reviews and Meta-Analyses guidelines for systematic reviews (12). We searched the electronic databases PubMed, Embase, MEDLINE, and the Cochrane Central Register of Controlled Clinical Trials for articles investigating HRV measurement in critically ill patients using combinations of the following key words: heart rate variability, sepsis, multiple organ dysfunction, critical illness, intensive care unit, shock, medication, and drug. The systematic search was overseen by a librarian and three adjudicators evaluated the tables for completeness.

Of the 28 studies identified, none rigorously incorporated medication exposure into HRV analysis (13–40). Approaches included exclusion of patients receiving medications known to influence HRV (e.g., beta-blockers), thereby avoiding confounding but reducing generalizability (18); descriptive reporting of medication use without analytic integration; or partial or inconsistent adjustment (e.g., treating all vasopressors as equivalent exposures regardless of agent or dose) (35). Additionally, no study incorporated time-varying medication covariates or interactions between medications and disease severity. **Supplemental Table 1** (https://links.lww.com/CCX/B606) illustrates the absence of meaningful medication integration across existing studies. This gap limits the validity, generalizability, and predictive accuracy of HRV as a clinical tool in real-world environments because medications are known to alter HRV (2, 8–10). For example, dobutamine, nesiritide, beta-blockers, and angiotensin-converting enzyme (ACE) inhibitors have all demonstrated measurable effects on HRV parameters in ICU populations (15, 25, 31, 34). Additional medications have been studied outside of critical illness, with their effects on HRV summarized in **Table 1** (13–15, 17, 25, 27, 28, 34–36, 41–47). Notably, many agents with potential autonomic or chronotropic effects (e.g., digoxin, amphetamines, amiodarone, bronchodilators, semaglutide) have yet to be studied, and their impact on HRV is still theoretical.

**TABLE 1.** Effects on Heart Rate Variability by Medications and Medication Class

Medication (Individual or Class)	Author, Year	Effect on HRV Variables in Patient Population
α_2_-agonists	Sevivas et al (36), 2024	No effect in HRV (in neonates)
Angiotensin-converting enzyme inhibitors	Schmidt et al (34), 2010	Increased HRV, especially very LF and total power
Beta-blockers	Hennen et al (25), 2008	Increased all HRV spectra
Carbamazepine	Leosuthamas et al (41), 2023	Decreased SDNN and HRV with significant decreases in HRV in sleep
Catecholamines (norepinephrine, dobutamine, epinephrine, and dopamine)	Schmidt et al (35), 2005	No change in HRV
Clonazepam	Leosuthamas et al (41), 2023	Decreased SDNN and HRV
Dexamethasone	Alonzo and Fairchild (14), 2017	Increased mean HRV and decreased HR characteristics index
Dobutamine	Aronson and Burger (15), 2001	Decreased HRV
	Nogueira et al (31), 2013	Negative correlation with LF alpha index (sympathetic activity)
Epinephrine	Jan et al (42), 2009	Decreased root mean square of successive differences of successive R-R intervals, percentage of successive normal RR intervals exceeding 50 ms, and HF
Fluoxetine	Kemp et al (43), 2016	No change in HRV
Hydrocortisone	Rassias et al (44), 2011	Increased approximate entropy
	Aboab et al (13), 2008	Increased LF_nu_–DBP
Hydrocortisone + fludrocortisone	Aboab et al (13), 2008	Increased LF_nu_–DBP and LF/HF ratio
Ivabradine	Kurtoglu et al (45), 2014	Increased all HRV spectra except for HR
Kampo	Zheng and Moritani (46), 2008	Decreased HR and increased the HF power
Levetiracetam	Leosuthamas et al (41), 2023	Decreased SDNN and HRV and decreased HF in sleep
Midazolam	Bradley et al (17), 2013	Increased HRV
High-dose nesiritide	Aronson and Burger (15), 2001	Decreased HRV
Low-dose nesiritide	Aronson and Burger (15), 2001	Increased HRV
Norepinephrine	Nogueira et al (31), 2013	Positive correlation with LF alpha index (sympathetic activity)
Pregabalin	Jiang et al (47), 2011	Decreased the LF/HF ratio
Propofol	Bradley et al (17), 2013	Increased HRV
Selective serotonin reuptake inhibitors (except fluoxetine)	Kemp et al (43), 2016	Decreased HRV

HF = high frequency, HR = heart rate, HRV = heart rate variability, LF = low frequency, LF/HF = low frequency to high frequency ratio, LF_nu_–DBP = normalized low frequency–diastolic blood pressure, SDNN = sd of the R-R intervals over a 24-hr period.

**Figure 1.**
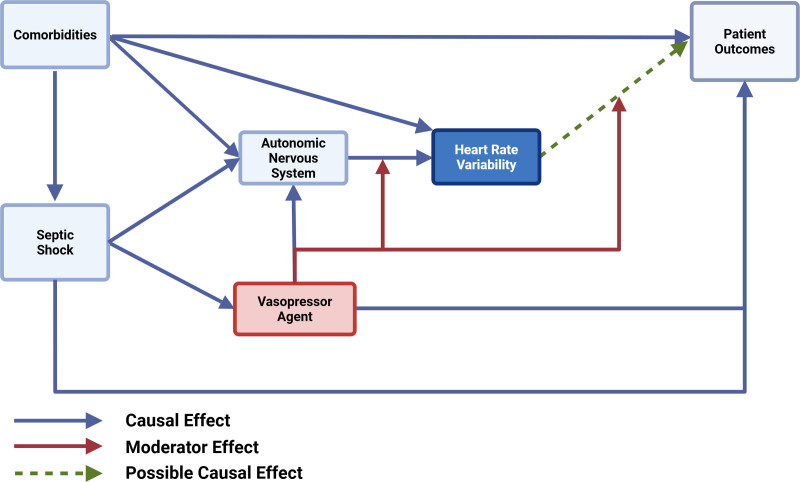
Directed acyclic graph of causal and moderating pathways in a simplified clinical scenario of a patient with septic shock on vasopressor therapy. Sepsis and comorbidities affect the autonomic nervous system (ANS), which, in turn, affects heart rate variability (HRV), which is associated with patient outcomes. Adding vasopressor medications complicates these interactions: vasopressors have their own independent effects on the ANS but may also moderate the effects of the ANS on HRV and the association between HRV and patient outcomes. This simple example highlights one acute problem and one medication class; the causal relationships become more complex with addition of fluid resuscitation, antibiotics, sedatives, corticosteroids, and other medications commonly administered to critically ill patients.

Critical illness, HRV, and medications are causally interconnected. HRV is influenced by multiple factors related to critical illness, including physiologic derangements such as those related to oxygenation and cardiac output, comorbidities, and supportive interventions such as mechanical ventilation. Meanwhile, medications represent a unique challenge because they can influence HRV through two distinct pathways that may overlap but are not necessarily related: direct modulation of autonomic tone and indirect effects mediated by improvement or worsening of the underlying disease process (**Fig. 1**). As a result, the impact of a medication on HRV is unlikely to be uniform but instead context dependent and varying across different disease states and different drug-disease pairings or multimedication, multidisease environments. For example, beta-blockers may alter HRV differently in sepsis than in heart failure because of their distinct interaction with each condition. In practice, critically ill patients are exposed to multiple overlapping causal factors determining HRV, making clinical interpretation of HRV indices highly complex. The relationships between medications and HRV must be more clearly delineated and incorporated into interpretation frameworks before HRV indices can be translated into reliable and clinically relevant monitoring tools.

## FUTURE DIRECTIONS

Addressing the gaps identified in current research will require study designs that explicitly measure, model, and interpret medication-associated changes in HRV rather than treating medication exposure as a confounder to be excluded. Pilot studies exploring feasibility and clarifying methodology will be essential to future work. To advance HRV toward meaningful clinical use, we propose several key research priorities.

### Quantify Medication Effects on HRV and Their Impact on Predictive Models

Medication-induced reductions or increases in HRV may reflect either worsening or improving clinical status. However, in some cases these medication-related interactions with HRV may instead represent noise and compromise its reliability and utility as a noninvasive biomarker for outcome prediction. For example, in one observational study a patient initiated on an epinephrine infusion experienced changes in HRV indicative of heightened sympathetic tone (48). This effect can be anticipated given the known pharmacological action of catecholamine vasopressors, and it would be ill-advised to avoid vasopressor support based solely on concerns surrounding HRV reduction. Still, the predictive value of HRV is clearly compromised in this scenario, and the effects of other medications on HRV and their clinical implications may not be as clear. Medication-induced HRV changes must be distinguished from disease-related changes to avoid misinterpretation and loss of predictive accuracy.

### Incorporate Medication Exposure Into HRV Prediction Modeling

When performing prediction modeling, excluding patients on medications that may impact HRV parameters avoids the complications presented by these significant confounders but limits generalizability. For example, an observational study demonstrating that HRV indices could predict sepsis approximately 60 hours before clinical diagnosis excluded all patients receiving beta-blockers and calcium channel blockers (18). In other prediction modeling approaches, medications were completely unaccounted for or patients with medications that could potentially impact HRV were excluded from the analysis (18, 19, 22, 23, 29, 30, 32, 33, 35, 40). Real-world utility of prediction models relies on representative cohorts, and as such studies excluding patients based on medication use or not accounting for medications must be interpreted with caution. However, it may be possible to improve both the generalizability as well as the predictive performance of HRV by using rigorous methods to account for medications and their directionality and magnitude of impact on HRV in prediction modeling approaches.

### Evaluate Whether Pharmacologically Influenced HRV Trends Reflect Treatment Response

While the effects of some medications on HRV may be related to direct effects on the autonomic nervous system and therefore represent artifactual changes, it is possible that other medications or treatments may have indirect effects on HRV via effects on an underlying disease process. It is unclear at this time if monitoring HRV after receiving some medications may be used to predict success or failure of that therapy. For example, if a patient with sepsis and low HRV due to the severity of illness receives appropriate broad-spectrum antibiotics and fluids, can an increase in HRV be indicative of appropriate selection of antibiotics and volume resuscitation? Does HRV trend correlate with other biomarkers that may be indicative of treatment success or failure (e.g., lactate)? A study in trauma patients found that HRV increased as base deficit decreased, thus highlighting its potential utility as a noninvasive measure of fluid resuscitation, but this finding has yet to be replicated in a larger, randomized study (22). An additional study in pediatric patients found that patients had a higher HRV on the last day of ICU admission compared with the first day, indicating that HRV may correlate with resolution of critical illness, but the generalizability of this study to a wider patient population is unclear (29).

### Clarify Whether HRV Is a Biomarker, a Pharmacologic Target, or Both

No studies to date have sought to determine an ideal or “target” HRV; however, if increased HRV is consistently shown to correlate with improved outcomes, there may be potential for using HRV as a goal to which pharmacologic interventions can be targeted. Furthermore, medications that increase HRV may warrant further research into their use for disease states associated with decreased HRV. For example, if ACE inhibitors are shown to increase HRV, potential roles in disease states characterized by low HRV (e.g., sepsis) may warrant investigation.

## CONCLUSIONS

HRV-based monitoring and prediction in critical illness holds substantial promise, and we posit that its clinical translation depends on rigorous characterization of medication effects in the context of disease states (49–53). Medications must not be treated as nuisance confounders but as integral physiologic modulators that must be incorporated into both interpretation and analytic models. We propose development of drug-specific and drug-disease state HRV adjustment indices, supported by advanced causal and statistical methods such as time-varying covariate models, marginal structural models, and propensity score analysis to further evaluate the interconnectedness of these concepts. Understanding how medications shape HRV is essential for transforming HRV from a promising research metric into a reliable tool for real-time clinical decision making in the ICU.

## ACKNOWLEDGMENTS

We thank Tara Kennell from the University of Georgia Library Services.

## Supplementary Material
