# Supplementary material for: Medication Effects on Heart Rate Variability in Critical Illness: The Overlooked Confounder
Source: Crit Care Explor. 2026 Mar 4;8(3):e1386. doi: 10.1097/CCE.0000000000001386 (PMC12962568; doi:10.1097/CCE.0000000000001386)
Supplement: Supplementary file 1 [file cc9-8-e1386-s001.pdf]

Supplemental Table 1. Summary of studies of HRV in critical illness

Author, Year	Patient population and setting	Study objective and design	HRV variables included	HRV measurement method	Adjustment for covariates (arrythmias), covariates in regression modeling, and adjustment for medications	HRV relationship to outcome
Sevivas, 2024	N= 16 neonates<br>42 assessments (intervention), 43 assessments (control)<br>Age: Neonatal<br>Location: NICU<br>Disease state: critically ill neonates requiring sedoanalgesia<br>Exclusion: end-of-life conditions, severe hypoxic-ischemic encephalopathies or cardiac arrhythmia	Objective: assess the impact of adjunct α2-agonist (dexmedetomidine or oral clonidine) administration on the measurement capacity of Newborn Infant Parasympathetic Evaluation (NIPE)<br><br>Design: observational, prospective, single-center study	HR-High Frequency oscillations (>0.15 Hz); HR, BP	NIPE monitor; NIPEm- calculates averaged over 20 min, NIPEi- calculates a mean value over 3 min	Arrythmia adjustment: none<br><br>Regression-modeling co-variates: model adjusted for confounders of postmenstrual age and pathology<br><br>Medication adjustments: none	α2-agonist administration does not affect the NIPE measurement capacity and therefore does not affect HRV in neonates.
Foroutan, 2019	N: 43<br>Age: adult<br>Location: ED or ICU<br>Disease state: trauma patients with a low injury severity score (ISS <24) and negative base excess on admission	Objective: Compare changes of HRV and BD during the treatment of trauma patients<br><br>Design: Prospective observational study<br><br>Methods: HRV and BD measured at admission, then after primary hydration (1L LR), and repeated when fluid resuscitation was terminated if patient required more fluid	HF, LF, HRV ratio (calculated to show changes in HRV from admission to after resuscitation)	Polar H7 HR sensor device was used to capture HR of patients, then using fast Fourier transform	Arrythmia adjustment: none<br><br>Regression-modeling co-variates: none<br>Medication adjustments: none	An increase in HRV may be a non-invasive index for the end point of resuscitation in trauma patients. A significant reverse correlation was found between BD ratio and the HRV ratio. At the time of BD clearance, there was an increase in HRV and a decrease in HRV after primary hydration had a significant connection with the need for ICU level care and transfusion of PRBCs.
Marsillio, 2019	N: 17<br>Age: Pediatric (0 to 18)<br>Location: pediatric ICU<br>Disease state: critical illness	Objective: identify whether changes in HRV could be detected as critical illness resolves<br><br>Design: Retrospective, observational study<br><br>Methods: HRV data collected and calculated during the first and last 24 hours of the PICU stay	RMSSD, % of successive NN interval differences over 50 ms (pNN50), and SDNN	HRV was calculated from the ECG waveforms obtained by BedMasterEx from the bedside monitor; to validate results from the real-time system, HRV values were compared to those obtained using Kubios	Arrythmia adjustment: Artifact was managed using an automated recursive procedure for detection of outliers<br><br>Regression-modeling co-variates: models adjusting for age were explored but did not affect the association between time from admission and log HRV in different time periods<br><br>Medication adjustment: none	RMSSD, pNN50, and SDNN are significantly lower in the first 24 hours of PICU stay when compared to the last 24 hours supporting the possibility of HRV to serve as a marker of clinical acuity; greater mean SDNN and pNN50 in the last 24 hours of PICU stay were significantly associated with greater PICU LOS
Alonzo, 2017	N= 109<br>Age: neonatal	Objective: determine whether HRV or its change after	HRV and HRC (heart rate	Continuous HRC index monitor (HeRO	Arrythmia adjustment: none	Dexamethasone administration for weaning off

	Location: NICU<br>Disease state: very low birth weight preterm infants receiving mechanical ventilation<br>Exclusion: surgery within 2 days of receiving dexamethasone, necrotizing enterocolitis	dexamethasone differed in infants who were successfully extubated compared to those who failed extubation<br><br>Design: retrospective analysis	characteristics) index (abnormal calculations of low HRV, transient decelerations, and low entropy)	monitor) via ECG signals	Regression-modeling co-variates: none<br><br>Medication adjustments: none	MV or for those who failed extubation cause a mean increase in HRV and decrease in the HRC index.
Brown, 2016	N: 95<br>Age: ≥ 15<br>Location: ICU<br>Disease state: septic shock who required treatment with vasopressors	Objective: Evaluate the relationship of HRV complexity before up-titration of vasopressor infusion rates and success of that increase<br><br>Design: Prospective observational study<br><br>Methods: Measurements of the complexity of HRV were taken 5 minutes before all vasopressor up-titrations in the first 24 hours of ICU admission	HR, MAP, fractal exponents of HRV	Phillips Intellivue Monitors were used to monitor HR and BP at 30-sec intervals and CIMVA was used to process the data in 5 min analytical windows; CIMVA complexity measures were also evaluated but were not transformed, combined, or modified	Arrythmia adjustment: none<br><br>Regression-modeling co-variates: APACHE II, SOFA, MAP, age, sex, change in norepinephrine equivalents<br><br>Medication adjustment: Vasopressors were adjusted to norepinephrine equivalents and included in the regression modeling	The long-term fractal exponent of HRV and MAP are associated with success of up-titration of a vasopressor and these variables should be considered when evaluating whether to up-titrate a vasopressor.
Huang, 2014	N= 101<br>Age: adult<br>Location: ICU<br>Disease state: critically ill requiring MV<br>Exclusion: arrythmias, chronic antiarrhythmic medications, resumed ventilatory support within 30 minutes of SBT, tracheostomies, unable to follow verbal instructions	Objective: investigate change of HRV during the entire weaning process in patients recovering from respiratory failure<br><br>Design: prospective observational study<br><br>Methods: performed between 8 AM and 12 PM using a 5-minute measurement of HRV	VLF, LF, HF, TP, LF/(TP-VLF) or HF/(TP-VLF) multiplied by 100, LF/HF	Embletta system to record electrocardiography pre-, during, and post-SBT periods	Arrythmia adjustment: excluded<br><br>Regression-modeling co-variates: age, sex<br><br>Medication adjustments: beta2 agonist, anticholinergics, CCB	Lower HRV (via TP) was significantly associated with SBT failure (p=0.003) with initial SBT and an inability to increase VLF and TP post-extubation correlated with extubation failure in those who passed their initial SBT (p=0.003 and p=0.004).
Zhang, 2014	N= 41 (intervention), X (control)<br>Age: adult<br>Location: ICU<br>Disease state: acute pancreatitis<br>Exclusions: chronic pancreatitis, pre-existing organ failure, received surgical treatment before admission, persistent	Objective: investigate the association between heart rate variability and disease severity in acute SAP and its prediction capabilities for IPN or MODS<br><br>Design: prospective observational study<br><br>Methods: 5-min ECG was obtained at bedside between 9:00	SDNN, RMSSD, VLF, LF, HF, normalized units of LF and HF, LF/HF	5 min ECG via a 12-lead electrocardiogram machine (EDAN)	Arrythmia adjustment: None<br><br>Regression-modeling co-variates: none<br><br>Medication adjustments: none	Lower LF and LF/HF measures were significantly different in those with MODS, IPN and non-survivors, while nHF was significantly higher in this group (p<0.01). LF/HF showed the highest specificity 94% and sensitivity 89% in predicting MODS.

	arrhythmia, cardiac pacemaker	and 11:00 within 24 hours of ICU admission				
Bradley, 2013	N= 21 patients; 31 sedation interruptions (SIs)<br>Age: adult<br>Location: ICU<br>Disease state: critically ill with respiratory and/or cardiac failure<br>Exclusion: arrythmias, transferred from aonther ICU	Objective: explore the effects of sedation on continuous HRV and respiratory rate variability (RRV) of ICU patients<br><br>Design: Prospective observational pilot study<br><br>Methods: computed over two periods—the 4 hrs prior to SI and the entire duration of SI by averaging the 5-min windows within each period	RRI, time domain (SD, probability distributions, frequency histograms), frequency domain (HF, LF, LF/HF ratio), time-frequency domain, entropy (approximate, sample, multiscale); scale-invariant (power law, detrended fluctuation analysis)	Continuous monitoring of their ECG at 125 Hz via the Philips Interllivue MP70 bedside monitors with a data cleansing process (RRI thresholds) and CIMVA scoring	Arrythmia adjustment: excluded, data cleansing process to remove artifacts and outliers<br><br>Regression-modeling co-variates: not adjusted<br><br>Medication adjustments: not adjusted	Greater HRV increase/restoration was seen in those receiving propofol rather than midazolam (however, more patients with higher MODS scoring patients were on midazolam). And a greater change in HRV was seen in patients with a low-medium MODS score than medium-high MODS score ($p < 0.05$).
Brown, 2013	N= 48<br>Age: adult<br>Location: ICU<br>Disease state: severe sepsis and septic shock<br>Exclusions: pregnant, admission DNR/DNI orders, non-sinus rhythm	Objective: Compare the complexity of HRV at ICU admission to determine the success of early resuscitation in sepsis patients<br><br>Design: prospective cohort study	Mean, number and proportion of consecutive NN50, sample entropy, Poincare plot SD, fractal exponents from detrended fluctuation analysis, power spectral density measurements including TP and power across HF, LF, and VLF bands	ECG via bedside Philips Intellivue monitors, 500 Hz for 6 hours following ICU admission	Arrythmia adjustment: excluded<br><br>Regression-modeling co-variates: adjusted for mean heart rate, APACHE II and SOFA scores, age, sex, exposure to vasopressors, presence of shock<br><br>Medication adjustments: none	Study suggests that fractional complexity, measured by the ratio of fractal alpha parameters, was associated with vasopressor independence at 24 hours and 28 day mortality.
Nogueira, 2013*	N= 31<br>Age: adult<br>Location: ICU<br>Disease state: severe sepsis or septic shock	Objective: evaluate vasoactive drugs on baroreceptor regulation and the interaction of this in regulation of HRV<br><br>Design: prospective observational study	HRV and blood pressure variability; low and high frequency	Not specified	Arrythmia adjustment: not specified<br><br>Regression-modeling co-variates: not specified<br><br>Medication adjustments: not specified	The use of dobutamine showed a negative correlation with the low-frequency alpha index (sympathetic activity); while norepinephrine showed a positive correlation.
Weiske, 2013	N= 71<br>15 (ICU-AW), 56 (no ICU-AW)<br>Age: Adult<br>Location: ICU<br>Disease state: critically ill, ICU-AW	Objective: investigate if critically ill patients with ICU-AW are more likely to have autonomic dysfunction than those without ICU-AW.	LF, HF, RR, total power, LF/HF ratio	10-minute continuous ECG monitor once daily, bedside monitor, sampling frequency, 250 Hz; high pass filter, 100 Hz	Arrythmia adjustment: excluded recordings with 20% artifact<br><br>Regression-modeling co-variates: age, MV, sedation status, SOFA score	There is no significant correlation between ICU-AW and autonomic dysfunction with ($p = 0.53$ and 0.75 for total power and LF/HF, respectively) or without ($p = 0.07$ and 0.36 for total power

	Exclusion: neuromuscular disorder, stroke, TBI, CNS infection, or CA as admission reason; previously autonomic neuropathy; elective post-surgical care; pre-hospital poor functional status or spinal injury	Design: prospective observational cohort study<br><br>Methods: HRV measured daily to a max of 15 days			Medication adjustments: norepinephrine, beta blocker, sedation	and LF/HF, respectively) covariate adjustment.<br><br>Confounders: percent of artifacts affected TP, LF, HF; while MV, sedation, NE dosage, and HR only associated with TP
Bravi, 2012	N: 17<br>Age: 46-62<br>Location: inpatient unit<br>Disease state: BMT for hematological malignancy or other disorder and developed neutropenia<br>Exclusion: pre-existing cardiopulmonary disease, receiving CCBs or BBs, pre-existing arrhythmia, contraindication to electrocardiogram adhesives	Objective: develop a composite measure of variability that can be used to identify and track sepsis development<br><br>Design: Prospective observational study<br><br>Methods: Continuous Holter ECG ~24 hrs before their BMT and continuing through their neutropenia resolution	SD, coefficient of variation, Power law Y intercept, detrended fluctuation analysis AUC, wavelet AUC, Shannon entropy, Plotkin-Swamy average energy, Fuzzy entropy, Correlation dimension global, cardiac vagal index, Largest Lyanupov exponent	A Zymed DigiTrak-Plus sampled the ECG at 175 Hz with 10-bit amplitude resolution, and annotated all normal QRS peaks and arrhythmias, including premature atrial and ventricular beats	Arrythmia adjustment: Premature beats were excluded<br><br>Regression-modeling co-variates: none<br><br>Medication adjustment: None but excluded patients taking BBs and CCBs	Researchers created a probability of developing sepsis using the HRV data. The composite revealed detected sepsis approximately 60 hours (median value) before sepsis diagnosis, although it was not a real-time system
Gomez Duque, 2012	N= 100<br>Age: adult<br>Location: ICU<br>Disease state: Sepsis with need for cardiovascular or respiratory support<br>Exclusions: clinical or ECG features complicating interpretation of the Holter recordings, CAD	Objective: describe the incidence of cardiovascular adverse events in the various stages of sepsis<br><br>Design: longitudinal, descriptive observational study<br><br>Methods: ECG monitoring from 6-48 hours following ICU admission	Standard deviation of SDNN and PNN50	48 hour Holter monitoring via Cardiovex MMC10L system	Arrythmia adjustment: none<br><br>Regression-modeling co-variates: none<br><br>Medication adjustments: none	The most common cardiovascular event that occurred in sepsis was arrythmias. Septic shock was associated with less HRV, more arrythmias and greater mortality than sepsis and sever sepsis categories. Those needing vasopressors and inotropes showed a greater loss of HRV.
Imabayashi, 2011*	N= 20<br>Age: not specified<br>Location: ICU<br>Disease state: treated on total mechanical ventilatory support<br>Exclusion: arrhythmia or pacemaker or other treatment during the measure time	Objective: investigated the effect of α_2-agonist, dexmedetomidine, on real-time heart rate variability<br><br>Design: prospective cohort study<br><br>Methods: HRV assessed before and 30 min after dexmedetomidine administration	HR, CV-RR, HF, LF/HF, SBP, CV-SBP, SBP-HF and SBP-LF/HF. The CV-RR was SD of RR intervals, and the CV-SBP was SD of systemic blood pressure	MemCalc system; 0.04 to 0.15 Hz (low frequency), 0.15 to 0.40 (high frequency)	Arrythmia adjustment: not specified<br><br>Regression-modeling co-variates: not specified<br><br>Medication adjustments: not specified	Administration of continuous dexmedetomidine decreased HR (p=0.017), CV-RR (p=0.085), and CV-SBP (p=0.038) but did not change HF, LF/HF, SBP-HF and SBP-LF/HF.

Schmidt, 2010	N=68 (intervention), 110 (control)<br>Age: adults<br>Location: ICU<br>Disease state: MODS with APACHE>20, intervention group received ACEi and control group did not	Objective: characterize benefits of ACEi therapy in MODS patients.<br><br>Design: Retrospective observational study	TP, VLF, LF, HF, and LF/HF ratio	24- hour continuous ECG recordings were taken using Holter recorders. The rate of ECG acquisition was 256 Hz. Artifacts and ectopic beats were removed and replaced with interpolated data. Only recordings with >20 hrs of usable data were included.	Arrythmia adjustment: none<br><br>Regression-modeling co-variates: APACHE II and SOFA<br><br>Medication adjustments: ACEi at admission, late ACEi, or no ACEi administration	ACEI administration decreased mortality and better preserved HRV (higher HRV than group that did not receive ACEI treatment), especially VLF and TP.
Tiainen, 2009	N= 70<br>36 (HT) vs 34 (NT)<br>Age: adult<br>Location: ICU<br>Disease state: witnessed CA from presumed cardiac origin<br>Exclusions: not responding to any verbal command after ROSC	Objective: examine the effects of therapeutic HT of 33°C after CA on cardiac arrhythmias and HRV and to evaluate their prognostic value<br><br>Design: prospective comparative substudy of RCT<br><br>Methods: Three recording for a 24-hour interval were obtained: 1. for 24 hours after randomization, 2. immediately after the first recording (hours 24–48), 3. 14 days after CA	HF, LF, TP, SDNN, SDANN,	24-hour ambulatory ECG monitoring via portable two-channel tape recorder (Oxford Medilog)	Arrythmia adjustment: none<br><br>Regression-modeling co-variates: treatment group, ROSC delay, age, occurrence of VTs during the first 2 days, EF on the second day TTE, etiology of the CA<br><br>Medication adjustments: none	During the first 24 hours after randomization, the HT group had all higher HRV measures than the NT group. The effect on SDNN and SDANN is significantly higher in the HT group through the 24-48 hours, but not TP, LF, HF.
Aboab, 2008	N = 81<br>Study 1: 5 (septic shock) vs 6 (healthy)<br>Study 2: 47 septic shock<br>N=23 septic shock with adrenal failure<br>Age: adults<br>Location: ICU<br>Disease state: septic shock<br>Exclusions: <18 yrs, nonsinus rhythm, pregnancy, acute MI, PE, previous corticosteroids, known autoimmune disease or immune suppression, chronic CV, pulmonary or neurologic diseases, DM, and other	Objective: determine the mechanism of the hemodynamic effects of hydrocortisone ± fludrocortisone in sepsis<br><br>Design: prospective, observational study<br><br>Methods: two 5 min recordings were performed 1 hour following hemodynamic stabilization; before and 60 minutes after hydrocortisone bolus; before and after corticotropin stimulation test; before and after initiation of scheduled corticosteroids or placebo	HF, LF, normalized HF and LF, area under the curve (AUC), LF/HF ratio, HR DBP	finger photoplethysmography method via Finapres 2300 device	Arrythmia adjustment: Excluded, removed artifact or ectopic beats<br><br>Regression-modeling co-variates: none<br><br>Medication adjustments: none	Septic shock patients had decreased LFnu-HR (0.16 ± .05 vs 0.23 ± .07 p = 0.01) and LFnu-DBP (0.18 ± 0.11 vs. 0.28 ± 0.02 p = 0.01) compared to healthy volunteers and had a greater increase in LFnu-DBP (p = 0.01) following a single hydrocortisone dose. Those with adrenal failure and septic shock had decreased LFnu-HR (0.1 ± 0.01 vs 0.2 ± 0.15 p = 0.01) and LFnu-DBP (0.008 ± 0.01 vs 0.14 ± 0.22 p = 0.0003) compared to normal adrenal function. Septic patients with adrenal failure receiving hydrocortisone ± fludrocortisone significantly increased LFnu-DBP (p = 0.02) and LF/HF ratio

	conditions associated with autonomic failure					compared to placebo (p = 0.009).
Hennen, 2008*	N: 157<br>Age: adults<br>Location: ICU<br>Disease state: MODS with APACHE II >20	Objective: determine if BB therapy could restore the interorgan communication and reduce mortality.<br><br>Design: Patients underwent 24 hours of ECG monitoring within the first 48 hr of admission. The mortality rate was assessed 28 days from admission. 69 patients received BB therapy compared to 88 patients who did not.	VLF, LF, HF, ln(VLF)	Sampling rate was 256 Hz	Arrythmia adjustment: not specified<br><br>Regression-modeling co-variates: not specified<br><br>Medication adjustments: not specified	BBs were associated with a higher survival probability (HR 0.4, 95% CI 0.23–0.68; p = 0.001). Survival benefit was especially seen in the subgroup of MODS patients who had an ischemically triggered MODS (HR 0.2 [0.1–0.5], p = 0.001). HRV was higher in patients who received BBs.
Nogueira, 2008	N= 31<br>Age: adult<br>Location: ICU<br>Disease state: severe sepsis and septic shock<br>Exclusion: arrythmias, acute/previous MI, permanent pacemaker, CHF functional class III or IV, DM	Objective: determine if HRV alteration in septic patients is determined or correlated to secondary cardiac damage from inflammation or metabolic changes<br><br>Design: prospective observational cohort study<br><br>Methods: 30-minute recordings of cardiac rhythm were obtained on the mornings of the days 1, 3, and 6.	Vasomotor low frequency, LF, HF, LF/HF ratio	30 minute Holter analysis was performed on days 1, 3, and 6. CardioFlash Digital Holter and CardioSmart Professional CSK 540 Holter software	Arrythmia adjustment: excluded if baseline arrythmia<br><br>Regression-modeling co-variates: none<br><br>Medication adjustments: none	Plasma FFA levels, LFnu index, troponin levels, and histological changes were all found to be correlated to each other with increased cardiac damage and non-survivors.
Passariello, 2007	N= 10 sudden death vs 30 (control)<br>Age: adult<br>Location: ICU<br>Disease state: anginal syndrome and ischemic sudden death<br>Exclusions: antiarrhythmic medications,	Objective: investigate the correlation between ischemic sudden death and autonomic nervous system activity<br><br>Design: Case-control study<br><br>Methods:	SDNN index, SDANN index and RMSSD, pNN50	24-hour Marquette Holter ECG monitoring	Arrythmia adjustment: artifacts, ectopic beats and significant ST segment changes were removed<br><br>Regression-modeling co-variates: gender and age matched, disease state matched<br><br>Medication adjustments: excluded those on antiarrhythmics and similar representation of antianginal therapies	Standard deviation NN was lower before ischemic sudden death occurred" epoch 3 54±12 ms (p< 0.005) and epoch 26±5 (p<0.005). SDNN in controls was 58±28 ms 5 min before the most significant episode of ST shift vs the sudden death group of 26±5 ms (p<0.001).
Bourgault, 2006	N= 18<br>Age: adult<br>Location: ICU<br>Disease state: critically ill patients receiving positive pressure ventilation	Objective: examine the autonomic mechanisms underlying HR and SBP responses to ETT suctioning and compare the open versus closed methods of ETT suctioning on these measures and on PaO2 in	LF peak, HF peak, HF/TP, LF/HF, RRI, SBP, SBR, TP, baroreflex sequences, Baroreflex slope	Marquette cardiac monitors, series 7010 and 8000, with peak R wave detectors before and after suctioning	Arrythmia adjustment: excluded<br><br>Regression-modeling co-variates: adjusted for gender, age, CAD, level of sedation, ETT size, respiratory rate, and tidal volume	No significant differences between open and closed suctioning methods on HRV and baroreflex measures.

	Exclusion: arrythmias, sympathomimetic, anticholinergic, or beta antagonists, heavy sedation, autonomic dysfunction	orally intubated patients receiving positive pressure ventilation<br><br>Design: prospective cohort study<br><br>Methods: assessments occurred before and after ETT suctioning interventions over 20 min			Medication adjustments: controlled for level of sedation, no heavy sedation	
Schmidt, 2005	N= 85<br>Age: adult<br>Location: ICU<br>Disease state: critically ill patients with MODS<br>Exclusion: nonsinus rhythm, inadequate recordings, intermittent pacemaker beats	Objective: investigate if autonomic function is reduced in MODS, if indexes of autonomic dysfunction are related to therapeutic interventions, if autonomic dysfunction is equally pronounced among different age groups, if the autonomic dysfunction seen in MODS has prognostic implications<br><br>Design: prospective observational cohort study<br><br>Methods: 24 hours continuous ECG and those with > 20 hours of recordings were included	PNN50, rMSSD, VLF, LF, HF, LF/HF ratio, SDNN, SDANN, baroreflex sensitivity, chemoreflex sensitivity	24 hours continuous ECG using Holter recorders at 256 Hz (DMS; MTM multitechmed GmbH; Ela Med)	Arrythmia adjustment: excluded, removed artifacts and ectopic beats<br><br>Regression-modeling co-variates: age, mechanical ventilation<br><br>Medication adjustments: sedation, catecholamines	There was no significant difference in autonomic function in those receiving sedation or catecholamines compared to those who were not. Different age groups had insignificant differences in HRV and chemoreflex but significant differences in baroreflex sensitivity. Mechanical ventilation did show lower frequency domain HRV measures, pNN50 and rMSSD. 28-day mortality was best predicted by lnVLF.
Chase, 2004	N; 5 (intervention), 13 (control)<br>Age: adult<br>Location: ICU<br>Disease state: critical illness (intervention) or healthy volunteers (control)<br>Exclusion: neuro-muscular blockade, head injury, or high morbidity.	Objective: quantify the correlation between HRV, BP, and BPV and agitation to determine if they can be used as markers to establish the patient's severity of agitation.<br><br>Design: prospective study<br><br>Methods: Patients were subjected to Stroop's color word test to induce mental stress and cold press test to induce physical pain. Blood pressure and heart rate data was recorded before and after the test. ICU received either morphine 1 mg/mL or midazolam 0.5 mg/mL for sedation and had it weaned off while monitoring agitation.	VLF/HF ratio	Marquette monitor and PC	Arrythmia adjustment: none<br><br>Regression-modeling co-variates: agitation<br><br>Medication adjustments: none	As morphine 1 mg/mL and Midazolam 0.5 mg/mL was weaned, patients became agitated. The Riker SAS showed good correlation with agitation.
Pontet, 2003	N= 11 MODS vs 11 non-MODS (control)<br>Age: adult<br>Location: ICU	Objective: determine whether HRV impairment preceded a diagnosis of MODS	SDNN, rMSSD, TINN, LF, HF, normalized units of LF and HF, LF/HF	Continuous ECG monitoring for 14 hours/day	Arrythmia adjustment: excluded<br><br>Regression-modeling co-variates: matching of age	SDNN and LF were significantly lower in the MODS group compared to the non-MODS group when

	Disease state: sepsis due to CAP or recent abdominal surgery<br>Exclusions: nonsinus rhythm, anticholinergics, beta-blocking medications	Design: prospective case control study<br><br>Methods: 5- and 60-min time segments (“epochs”)			between the MODS and non-MODS groups<br><br>Medication adjustments: excluded anticholinergic and beta blockers	adjusted for age, 5.5 ms ± 3.6 vs 11.4 ms ± 7.6 and 11.3 ms ±16.3 vs 108.8 ms ± 199.5 respectively (p=0.047 and p=0.13). LF seemed to be the best predictor of MODS in the multivariant regression model.
Shen, 2003	N= 24<br>Age: adult<br>Location: ICU<br>Disease state: critically ill<br>Exclusion: arrhythmias, already or ready to be tracheostomized	Objective: investigate the change of autonomic nervous activity during ventilator weaning by HRV analysis<br><br>Design: prospective observational cohort study<br><br>Methods: HRV was measured in three phases: assist/control mandatory ventilation (ACMV), PSV, and SBT	LF, HF, TP, normalized unit of HF and LF, LF/HF ratio	three-channel ambulatory ECG recorder via Del Mar 563	Arrythmia adjustment: excluded<br><br>Regression-modeling co-variates: none<br><br>Medication adjustments: none	Weaning mechanical ventilation from a PSV setting to SBT resulted in significant decreases in HRV measures in the failure group (TP, p = 0.025; LF, p = 0.007; HF, p = 0.031), but not in the success group.
Aronson, 2001	N: 199 (46 clinical sites)<br>Age: adult<br>Location: ICU<br>Disease state: Class II or IV NYHA heart failure	Objective: determine whether use of BBs improves HRV during decompensated heart failure<br><br>Design: randomized trial comparing nesiritide to dobutamine	Total power spectrum and high frequency, low frequency, very low frequency, and ultra -low frequency power	24-hour Holter monitoring before administration of drugs. Holter recordings were obtained and analyzed using a 2010, Zymed Medical Instruments scanner.	Arrythmia adjustment: Research analyzed ventricular tachycardia events, defined as ≥3 premature ventricular beats at >100 bpm<br><br>Regression-modeling co-variates: Age, sex, DM, etiology of heart failure (ischemic or non-ischemic), serum sodium, serum creatinine, blood pressure, and medications<br><br>Medication adjustment: Multivariate analysis included digoxin, ACEi, ARBs, vasodilators, and antiarrhythmics	Beta blockade had a positive correlation with HRV overall and a positive correlation with each spectra component as well, although doses of nesiritide and dobutamine were not recorded
Korach, 2001	N= 41<br>Age: adults<br>Location: ICU<br>Disease state: critically ill<br>Exclusions: nonsinus rhythm, drug poisoning, cardiogenic pulmonary edema, permanent pacemaker, transferred from other ICU	Objective: identifying the factors associated with impaired sympatho-vagal balance in critically ill patients<br><br>Design: prospective cohort study<br><br>Methods: 30-min record of cardiac rhythm was obtained in the morning between 8 and 12am	HRV, LF, HF, VLF, LF/HF ratio	Numeric acquisition system BHL6000 obtained for 30-min for cardiac rhythm monitoring, sample rate of 5Hz	Arrythmia adjustment: excluded<br><br>Regression-modeling co-variates: age, sepsis, SAPS II<br><br>Medication adjustments: catecholamines, sedation	Those with a low LF/HF ratio were more likely older, septic, high SAPS II score, receiving catecholamines and sedation. Following adjustments only age and sepsis was associated with a lower LF/HF ratio and higher risk of mortality is less than 1.5 (OR 3.63; 95% CI: 1.47-9.01, p=0.005).

Garrard, 1993	N: 17<br>Age: 21-83<br>Location: ICU<br>Disease state: sepsis syndrome<br>Exclusion: PMH of DM, CKD, HTN, or cardiac disease	Objective: Determine the contributions of sympathetic and parasympathetic activity to HRV in patients with sepsis<br><br>Design: Prospective observational study<br><br>Methods: HRV was measured during sepsis and then again after recovery	High frequency (referred to as respiratory frequency power (RFP)) and low frequency power (LFP)	Raw ECG data was taken using three electrodes on chest wall and was processed to detect QRS complex using an 8088 processor and 8078 math co-processor.	Arrythmia adjustment: none<br><br>Regression-modeling co-variates: APACHE II and TISS<br><br>Medication adjustments: No adjustment. All patients received sedative agents at a range that allowed for response to verbal commands and beta agonists.	Sympathetically mediated heart rate variability decreases with sepsis severity (p<0.0001). Total HRV decreases with sepsis severity (p<0.0005)

ACEi: angiotensin converting enzyme inhibitor; ACMV: assist/control mandatory ventilation; ANN: average of normal to normal intervals, mean of all RR intervals; APACHE: Acute Physiology and Chronic Health Evaluation II; ARB: angiotensin receptor blocker; ARNi: angiotensin receptor/neprilysin inhibitor; AUC: area under the curve; BB: beta blocker; BD: base deficit; BMT: bone marrow transplant; BP: blood pressure; bpm: beats per minute; BPV: blood pressure variability; CA: cardiac arrest; CAD: coronary artery disease; CAP: community acquired pneumonia; CCB: calcium channel blocker; CHF: chronic heart failure; CI: confidence interval; CIMVA: Continuous Individualized Multiorgan Variability Analysis; CKD: chronic kidney disease; CNS: central nervous system; CV: cardiovascular; CV-RR: standard deviation of RR intervals; CV-SBP: standard deviation of systemic blood pressure; DBP: diastolic blood pressure; DM: diabetes mellitus; DNI: do not intubate; DNR: do not resuscitate; ECG: electrocardiogram; ED: emergency department; EF: ejection fraction; ETT: endotracheal tube; FFA: free fatty acid; HF: high frequency; HR: hazard ratio; hr: hour; HRC: heart rate characteristics; HRV: heart rate variability; HT: hypothermia; HTN: hypertension; Hz: Hertz; ICU: intensive care unit; ICU-AW: intensive care unit-acquired weakness; IPN: infected pancreatic necrosis; ISS: injury severity score; IV: intravenous; L: liter; LF: low frequency; LF_{nu}: low frequency normalized for area under the curve; LFP: low frequency power; LOS: length of stay; LPS: lipopolysaccharide; LR: lactated ringers; MAP: mean arterial pressure; mg: milligram; MI: myocardial infarction; mL: milliliter; MODS: multiple organ dysfunction syndrome; MRA: mineralocorticoid receptor antagonists; MV: mechanical ventilation; NE: norepinephrine; nHF: normalized high frequency; NICU: neonatal intensive care unit; NIPE: newborn infant parasympathetic evaluation; NIPEm: mean NIPE value over 20 minutes; NIPEi: mean NIPE value over 3 minutes; NN: the time between two successive normal beats; NN50: number of consecutive normal-to-normal intervals that differ by more than 50milliseconds; NREM: non-rapid eye movement; NS: normal saline; NSR: normal sinus rhythm; NT: normothermic; NYHA: New York Heart Association; OR: odds ratio; PE: pulmonary embolism; PC: personal computer; PICU: pediatric intensive care unit; PMH: past medical history; pNN50: percentage of consecutive normal-to-normal intervals that differ by more than 50 milliseconds; PRBC: packed red blood cell; PSV: pressure support ventilation; RCT: randomized control trial; RFP: respiratory frequency power; RMSSD: root-mean-square successive difference; ROSC: restoration of spontaneous circulation; RR: time between two consecutive R waves; RRI: R-R interval; RRV: respiratory rate variability; RSA: respiratory sinus arrhythmia; SAP: severe acute pancreatitis; SAS: sedation-agitation scale; SBP: systolic blood pressure; SBT: spontaneous breathing trial; SD: standard deviation; SDANN: the standard deviation of 5-min RR intervals; SDNN: standard deviation of normal-to-normal intervals; SDDNN: standard deviation of the difference between normal-to-normal intervals; SGLT2i: sodium glucose-linked transporter 2 inhibitor; SOFA: Sequential Organ Failure Assessment; SSRI: selective serotonin reuptake inhibitor; SUDEP: sudden unexpected death in epilepsy; TBI: traumatic brain injury; TINN: triangular interpolation of R-R interval; TISS: therapeutic intervention scoring system; TP: total power; TSST: Trier Social Stress Test; TTE: transthoracic echocardiogram; VLF: very low frequency; VT: ventricular tachycardia;

*Available only as an abstract so data is limited
